# Supplementary material for: Assessing trauma center accessibility in the Southeastern region of the U.S. to improve healthcare efficacy using an anti-covering approach
Source: PLOS Glob Public Health. 2023 Aug 18;3(8):e0002230. doi: 10.1371/journal.pgph.0002230 (PMC10437900; doi:10.1371/journal.pgph.0002230)
Supplement: Attachment — Submitted filename: Response_to_Reviewers.docx [file pgph.0002230.s001.docx]

30 Apr 2023

Sub. Submission of authors’ response to reviewers’ comments and the revised manuscript titled " **Assessing trauma center accessibility in the Southeastern region of the U.S. to improve healthcare efficacy using an Anti-covering approach**" for publication in *PLOS One Global Public Health*

Dear Editors,

Thanks for the opportunity to revise our paper titled " **Assessing trauma center accessibility in the Southeastern region of the U.S. to improve healthcare efficacy using an Anti-covering approach**" We are grateful to reviewers for giving us valuable insights and comments on our original submission. We have reviewed the manuscript and revised all the issues and concerns indicated by the reviewers. We hope you and the reviewers will like what we have updated after the revision.

I summarize below the major points of the revisions:

1. **[Editors’ Comment 1]** We have thoroughly proofread the manuscript to address grammatical errors, unclear syntax, and problematic sentence structures. We carefully revised multiple grammatical errors and focused on enhancing readability by correcting the structure of erroneous sentences.
2. **[Editors’ Comment 2]** We have sorted the data in tables based on their respective key field values and added notes to identify the key fields for each table, Tables 1, 2, 3, and 4.
3. **[Editors’ Comment 3]** We have replaced the low-resolution figures (Figures 3, 4, 5, 6, and 7) with newly prepared sharp images for this revision.
4. In the method section, we have included brief paragraphs introducing location problems to clarify the methodology's features to the average reader and researchers. (p.7)
5. In the concluding remark, we included the limitations of the study and suggestions for future research. Furthermore, policy recommendations were provided to encourage collaboration among various EMS-related stakeholders and researchers.
6. Limitations of the study and future studies were added to the conclusion. Also, the recommendation was presented so various stakeholders could cooperate in archiving the database and developing the model.
7. We meticulously reviewed the reference list and found a reference ([23]) without year and page information. We included the year and page information in the reference [23]. (p.22) We have reviewed the citations and references format according to *PLOS One Global Public Health*’s format and style guidelines. (pp.20-22)

In the following section, please see how we have addressed every comment of each reviewer.

**Response to the first reviewers’ comments**

**Point 1:** However, from the early abstract to the methods, the TraCt model methods are not clarified to the uninitiated, lay global health reader without a background in statistics, or in previous literature. ACLP for a trauma center location problem should be clearly defined and described in the methods. Clarity can be added through the text and more needs to be done in clearly defining the modeling that was used earlier in the manuscript, and explaining some background to the modeling to ground this in content clarified this paper, beyond a reference.

**Response 1:**

In the method section, we appended summary paragraphs describing the unique features of diverse location problems to introduce the origin of the novel approach of this research. (p.7) We expect this part will help both general readers and researchers gain a better understanding of the location problem and its context chosen for this research.

**Response to the second reviewers’ comments**

**Point 1:** The article presents a well-structured and coherent narrative of the empirical results and policy implications of the study. However, it could benefit from a more nuanced approach that emphasizes the key contributions and future directions of the research.

**Response 1:** In the concluding remark, we included the limitations of the study and suggestions for future research. We expect that these structural reinforcements will highlight the key contributions of this study. Through this study, the blueprint for future research that can increase the possibility of more practical application has become clear. (p.18-19)

**Point 2:** To this end, it is crucial to highlight the innovative approach of the Anti-covering location problem (ACLP) used to assess trauma center accessibility, which represents a significant step forward in the field of healthcare efficacy.

**Response 2:** We added summary paragraphs describing the unique features of diverse location problems to introduce the origin of the novel approach of this research. (p.7) We expect this part will help general readers and professional researchers find the innovative utility of the TraCt model among the diverse location problems. Specifically, the TraCt model can be employed to assess the spatial disparity in accessibility and find the optimal location of facilities.

**Point 3:** Moving on to the most significant findings of the study, it is crucial to underline the potential demand that remains unaddressed in the Southeastern region of the U.S. The identification of optimal locations of trauma centers through the TraCt model and the subsequent reduction of spatial inequality in accessing trauma centers represent a critical achievement that cannot be overstated.

However, it is important to acknowledge the limitations of the study and explore future directions for research that can enhance its impact. For instance, incorporating more data sources and analytical techniques, considering other factors such as socio-economic status and transportation infrastructure, and expanding the spatial extent of the study area are all viable options that could significantly improve the research's overall value.

**Response 3:** We described the limitations of the study in terms of data availability and the model itself as a current static situation. Based on the contemplation regarding study limitations, we derived future research directions to improve the model’s reality and consider the spatiotemporal dynamics in EMS and trauma care services. (p.18-19)

**Point 4:** Lastly, practical recommendations for policymakers and healthcare professionals must be presented based on the study's findings. Highlighting the need for cooperation between county governments to transfer trauma patients to trauma centers in a timely fashion, utilizing the TraCt model to identify optimal locations of trauma centers, and emphasizing the importance of considering the spatial extent of the study area in addressing spatial disparities in accessing trauma centers are all crucial steps that can be taken to improve healthcare efficacy in the region.

**Response 4:** At the end of this manuscript, we highlighted the importance of active coordination among diverse agents, and anyone related to the trauma care or healthcare industries, especially in data sharing and collaboration for model development. (p.19)

We sincerely hope we have addressed all of your and reviewers’ concerns.

Please let me know if we need to revise anything else.

Best regards,

Authors

**Heewon Chea^*^**

Center for Transportation Research

University of Tennessee, Knoxville

Knoxville, TN 37996

[hchea@vols.utk.edu](mailto:hchea@vols.utk.edu)

** Corresponding author*

**Hyun Kim**

Department of Geography

University of Tennessee, Knoxville

Knoxville, TN 37996

[hkim56@utk.edu](mailto:hkim56@utk.edu)
