# Supplementary material for: Assessing trauma center accessibility in the Southeastern region of the U.S. to improve healthcare efficacy using an anti-covering approach
Source: PLOS Glob Public Health. 2023 Aug 18;3(8):e0002230. doi: 10.1371/journal.pgph.0002230 (PMC10437900; doi:10.1371/journal.pgph.0002230)
Supplement: Attachment — Submitted filename: Response_to_Reviewers.docx [file pgph.0002230.s002.docx]

24 June 2023

Sub. Submission of authors’ response to editors’ comments and the revised manuscript titled **“Assessing trauma center accessibility in the Southeastern region of the U.S. to improve healthcare efficacy using an Anti-covering approach”** for publication in *PLOS One Global Public Health*

Dear Editors,

I appreciate your comments to improve the quality of our paper titled **“Assessing trauma center accessibility in the Southeastern region of the U.S. to improve healthcare efficacy using an Anti-covering approach.”** We have reviewed the manuscript again and revised all the issues and concerns indicated by the editors. We hope you like the updated manuscript after the revision.

I summarize below the major points of the revisions:

1. We thoroughly reviewed the citations and the reference list (pp.20-22) and revised the broken links, which did not work (p.21 [13], [17]) or were directed to the wrong destination (p.20 [8]). I replaced the reference [8] because I found a better reference published more recently. We have revised the reference format following *PLOS One Global Public Health*’s format and style guidelines (p.21 [9]).
2. We have proofread the manuscript and carefully revised minor grammatical errors and typos.

There were no specific comments from reviewers in this cycle of revision.

We sincerely hope we have addressed all of your concerns.

Please let me know if there is anything else we need to revise.

Best regards,

Authors

**Heewon Chea^*^**

Center for Transportation Research

University of Tennessee, Knoxville

Knoxville, TN 37996

[hchea@vols.utk.edu](mailto:hchea@vols.utk.edu)

** Corresponding author*

**Hyun Kim**

Department of Geography

University of Tennessee, Knoxville

Knoxville, TN 37996

[hkim56@utk.edu](mailto:hkim56@utk.edu)
